# Growth Differentiation Factor 15 (GDF-15) Plasma Levels Increase during Bleomycin- and Cisplatin-Based Treatment of Testicular Cancer Patients and Relate to Endothelial Damage

**DOI:** 10.1371/journal.pone.0115372

**Published:** 2015-01-15

**Authors:** Renske Altena, Rudolf S. N. Fehrmann, Hink Boer, Elisabeth G. E. de Vries, Coby Meijer, Jourik A. Gietema

**Affiliations:** 1 Department of Medical Oncology, University of Groningen, University Medical Centre Groningen, Groningen, the Netherlands; 2 Department of Genetics, University of Groningen, University Medical Centre Groningen, Groningen, the Netherlands; University of Algarve, PORTUGAL

## Abstract

**Introduction:**

Chemotherapy-related endothelial damage contributes to the early development of cardiovascular morbidity in testicular cancer patients. We aimed to identify relevant mechanisms of and search for candidate biomarkers for this endothelial damage.

**Methods:**

Human micro-vascular endothelial cells (HMEC-1) were exposed to bleomycin or cisplatin with untreated samples as control. 18k cDNA microarrays were used. Gene expression differences were analysed at single gene level and in gene sets clustered in biological pathways and validated by qRT-PCR. Protein levels of a candidate biomarker were measured in testicular cancer patient plasma before, during and after bleomycin-etoposide-cisplatin chemotherapy, and related to endothelial damage biomarkers (von Willebrand Factor (vWF), high-sensitivity C-Reactive Protein (hsCRP)).

**Results:**

Microarray data identified several genes with highly differential expression; e.g. Growth Differentiation Factor 15 (*GDF-15*), Activating Transcription Factor 3 (*ATF3*) and Amphiregulin (*AREG*). Pathway analysis revealed strong associations with ‘p53’ and ‘Diabetes Mellitus’ gene sets. Based on known function, we measured GDF-15 protein levels in 41 testicular patients during clinical follow-up. Pre-chemotherapy GDF-15 levels equalled controls. Throughout chemotherapy GDF-15, vWF and hsCRP levels increased, and were correlated at different time-points.

**Conclusion:**

An unbiased approach in a preclinical model revealed genes related to chemotherapy-induced endothelial damage, like *GDF-15*. The increases in plasma GDF-15 levels in testicular cancer patients during chemotherapy and its association with vWF and hsCRP suggest that GDF-15 is a potentially useful biomarker related to endothelial damage.

## Introduction

The introduction of platinum-based chemotherapy in the late seventies has resulted in high cure rates in patients with metastatic testicular cancer. However, this chemotherapy causes side-effects, resulting in morbidity in successfully treated survivors [1]. Relevant issues are the increased risks for second malignancies and cardiovascular disease (CVD) [2–5]. CVD can arise during or shortly after treatment [6, 7], as well as years to decades later [3–5, 8–11].

One of the mechanisms involved in the development of this treatment-related CVD is direct endothelial damage. In addition to induction of endothelial cell death [12–14], both bleomycine [14–16] and cisplatin [14, 17–19] indirectly influence endothelial cell function, e.g. through interference with inflammatory and fibrinolytic factors. Ultimately, this chemotherapy-induced cellular activation can progress to endothelial dysfunction, accelerated atherosclerosis and overt CVD.

Early recognition and possibly prevention of these treatment-related complications are critical to maintain an optimal health condition of testicular cancer survivors. The development of CVD is a gradual process, and early interventions or intensified screening may slow down or stop the progression towards overt clinical morbidity. Biomarkers for treatment-related endothelial damage can identify those patients at increased risk for CVD.

In this study we back-translated the clinical finding that bleomycin and cisplatin induce endothelial damage. We used an unbiased approach by analysing cDNA microarray results to identify novel genes associated with chemotherapy-related endothelial damage. Gene expression profiles were generated from the human microvascular endothelial cell line (HMEC-1) before and after treatment with bleomycin and cisplatin at different time points and concentrations. Quantitative Real Time PCR (qRT-PCR) was performed to confirm genes with significant expression differences in several experimental settings. Next, based on known function of these genes in the literature, we selected one of these candidate genes for further validation as proof of principle at the protein level in a testicular cancer patient cohort. With this translational approach we aimed to identify mechanisms of and potential biomarkers for chemotherapy-related endothelial damage.

## Materials and Methods

### Cell line model

HMEC-1 is an immortalised human dermal micro-vascular endothelial cell line that retains its morphologic and functional endothelial cell characteristics during several passages [20]. Cells were grown as a monolayer in MCDB-131 medium (Invitrogen, Merelbeke, Belgium) supplemented with 10% foetal calf serum (Bodinco, Alkmaar, the Netherlands), 10 mM L-glutamine (Invitrogen, Merelbeke, Belgium), 1 μg/mL hydrocortisone (Sigma-Aldrich, Amsterdam, the Netherlands) and 10 ng/mL human epidermal growth factor (R&D Systems, Abingdon, UK), and were cultured at 37°C in a humidified atmosphere containing 5% CO_2_. Experiments were performed between passages 15–30.

In an “acute”-exposure setting, HMEC-1 were left untreated as controls, or were treated with 0.3 (IC_50_ (concentration inhibiting cell survival by 50%)) or 1.5 μg/mL (IC_90_) bleomycin and 2.6 (IC_50_) or 12.9 μM (IC_90_) cisplatin for 6, 24, and 48 hours (S1A Fig.). The IC50 values for both bleomycin and cisplatin fall within the plasma physiological concentrations of these drugs in patients during active treatment [14, 21–22]. In addition, in a “chronic”-exposure setting, lower doses were administered (IC_10_; bleomycin 0.06 μg/mL or cisplatin 0.52 μM) two times a week; cells were collected for analysis at day 30 (S1B Fig.). Administration of cisplatin had to be withheld at the 7^th^ administration because of considerable cell death, but was continued at full dose thereafter. Bleomycin could be administered without interruption.

### cDNA microarray experiments

Total RNA was isolated from HMEC-1 by a RNeasy kit (Qiagen, Venlo, the Netherlands) and pooled for each time-point and drug from 2 independent experiments. After purification (Qiaquick PCR purification kit, Qiagen, Venlo, the Netherlands), amplified RNA (cRNA) samples were transformed to cDNA with reverse transcriptase, independently labelled with Cy3 (green) and Cy5 (red), and randomly hybridised to the custom-made 18K cDNA microarrays. Fluorescent images of the microarray slides were obtained with the Affymetrix GMS428 scanner (Affymetrix, Santa Clara, CA) for both fluorophores, signal intensities for each spot were quantified by dedicated IMAGENE 5.6 software (Biodiscovery, Marina del Rey, CA).

Quantile normalisation was applied to log2 transformed Cy3 and Cy5 intensities. Operon v2.0 (Human Genome Oligo Set V2) probe identifiers were converted to official HUGO gene symbols. Expression values of multiple probes targeting a single gene were averaged, resulting in a total of 15,950 unique genes. Subsequently, expression data obtained from multiple hybridisations (*n* = 4) of the same HMEC-1 specimen were averaged.

The data have been deposited in NCBI’s Gene Expression Omnibus (GEO) and are accessible through GEO Series Accession number GSE62523 (http://www.ncbi.nlm.nih.gov/geo/query/acc.cgi?acc=GSE62523).

### Class comparison

In the “acute” exposure setting, differentially expressed genes between HMEC-1 untreated samples and samples exposed to the different drug dosages (i.e. IC_50_ and IC_90_) were tested with the non-parametric Cuzick test for linear trend, resulting in a *Z* score and a *P* value. This test was done for cells collected after 6 (*t* = 6), 24 (*t* = 24) and 48 (*t* = 48) hours. For all three time-points the *Z* score resulting from the Cuzick tests for linear trend was summed (i.e. *ΣZ* = *Z_t = 6_* + *Z_t = 24_* + *Z_t = 48_*); thereby selecting on genes with the constraint that changes in expression in a consistent direction with increasing concentrations over time. Genes were ranked according to their *ΣZ* score.

In the “chronic” exposure setting, a T-test was performed on gene expression levels obtained from samples exposed to the drugs (IC_10_ cisplatin or IC_10_ bleomycin) versus the untreated control samples that were collected after 30 days incubation. Results of these genes were ranked according to *P-*value.

### Gene Set Enrichment Analysis

Gene Set Enrichment Analysis (GSEA) [23] was executed with GSEA 2.0 software package (Broad Institute, Cambridge, MA). Expression data of all 15,950 genes were compared against functional gene sets to determine whether any of these sets were enriched in HMEC-1 treated with bleomycin or cisplatin in the “acute” as well as the “chronic” exposure setting. The comparison was performed using 169 gene sets from Kyoto Encyclopedia of Genes and Genomes database (KEGG; http://www.genome.jp/kegg/). Statistical significance of enrichment was determined using an empirical gene-based permutation test using 1000 permutations. A false discovery rate (FDR) was calculated for each functional gene set, which represent the estimated probability that a given enrichment score represents a false positive finding. We report gene sets with a FDR ≤ 0.10 and *P* ≤ 0.025.

### Quantitative Real Time PCR

Differential expression of three genes was validated by qRT-PCR. For this purpose, RNA samples included in the cDNA microarray analysis were used. In addition, two independent experiments were performed in which HMEC-1 was exposed to cisplatin and bleomycin according to the “acute”-exposure setting. RNA samples were isolated after 6, 24 and 48 hours exposure to the drugs (S1A Fig.). All RNA samples were DNase treated to eliminate genomic DNA-contamination, and subsequently, RNA was reverse transcribed into cDNA. qRT-PCR was performed using Applied Biosystems TaqMan assays, according to the manufacturers protocol. Master Mix, primers and TaqMan probes were purchased from Applied Biosystems (Nieuwerkerk a/d IJssel, the Netherlands). Three genes, with highly differential expression in three out of four experimental settings, were considered plausible candidates for qRT-PCR validation. The genes and their respective Taqman gene expression assay numbers were Growth Differentiation Factor 15 (*GDF-15;* Hs00171132_m1), Activating Transcription Factor 3 (*ATF3;* Hs00231069_m1), Amphiregulin (*AREG;* Hs00155832_m1); in addition expression of the housekeeping gene Glyceraldehyde 3-phosphate dehydrogenase (*GAPDH*; Hs02758991_g1) was determined. All experiments were performed in triplicate using ABI PRISM 7900 HT Sequence Detection System, with the following cycling conditions: 2 min at 50°C, 10 min at 95°C, followed by 40 cycles of 15 sec at 95°C and 1 min at 60°C. Relative quantity of target genes was calculated by dividing the mean cycle threshold (CT) for the gene of interest by the mean CT-value for the housekeeping gene *GAPDH*. Relative expression-differences were calculated by comparing expression to the baseline time-point (t = 6, S1A Fig.). Two-sided t-test was used to compare differences in expression. P-values < 0.05 were considered to indicate a significant difference.

### GDF-15 protein levels in testicular cancer patient plasma during and after bleomycin- and cisplatin-based chemotherapy

To clinically validate the findings from the cell line model, we used a cohort of 41 testicular cancer patients who participated in a prospective study on early chemotherapy-related cardiovascular changes during bleomycin- and cisplatin-based regimens. Patients eligible for the study had metastatic testicular cancer, were 18–50 years old and were receiving first line cisplatin-based chemotherapy at the University Medical Centre Groningen, the Netherlands. Exclusion criteria were previous chemotherapy or radiotherapy, presence of CVD, use of erythropoietin and glomerular filtration rate < 60 mL/min. The local ethics committee approved the study, and written informed consent was obtained from all participants. Depending on their International Germ Cell Cancer Collaborative Group (IGCCCG) prognosis group, patients received either three or four BEP courses lasting 3 weeks each (bleomycin—30 USP, days 2, 8 and 15; etoposide—100 mg/m^2^, days 1–5) and cisplatin—20 mg/m^2^, days 1–5). During the first 6 days patients were hydrated with 4 L NaCl 0.9%/day and received daily anti-emetic therapy (dexamethason, ondansetron). Blood samples were drawn at day 1, 8 and 15 of the first chemotherapy course, day 1 and 8 of the second and third course, (c1d1 (= baseline), c1d8, c1d15, c2d1, etc.), one month after completion and one year after start of chemotherapy. EDTA plasma was serially collected and stored in -20°C until analysis. Reference data were obtained from healthy male siblings of adult childhood cancer survivors, who had participated as control subjects in a cross-sectional study on late cardiovascular sequelae of treatment for childhood cancer [24]. Out of these healthy male siblings, a control group with a comparable median age as the testicular cancer patients was selected. Measurements in the controls were performed as described above.

Plasma GDF-15 protein levels were determined by sandwich enzyme-linked immunosorbent assay (ELISA) with a commercially available kit (R&D Systems, Abingdon, UK). Furthermore, these GDF-15 protein levels were related to plasma markers for endothelial damage (von Willebrand Factor (vWF), measured as described earlier) [25] and systemic inflammation (high-sensitivity C-Reactive Protein (hsCRP), as described earlier) [26]. For analysis of changes in these markers, non-normally distributed data are represented as median (range). For comparisons between groups the non-parametric Mann-Whitney U test was applied; the Wilcoxon’s signed rank test was used for paired changes. Two-sided P-values ≤ 0.05 were considered to indicate significance, SPSS software package version 22 (SPSS Inc., Chicago, IL) was used.

## Results

### cDNA microarray

**Figure 1:**
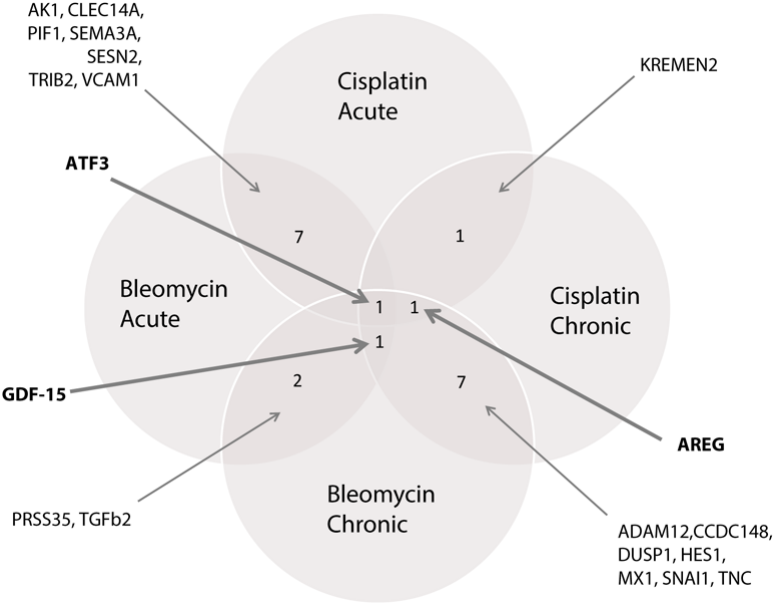
Overlapping genes in top 50 of most differentially expressed genes in HMEC-1 exposed to bleomycin and cisplatin.

**Class comparison.** The top 50 of most differentially expressed genes in the “acute” and “chronic” exposure setting for bleomycin and cisplatin are summarised in Table 1. Fig. 1 shows a Venn diagram of the overlapping genes in the top 50’s of the four different exposure settings. From this analysis three genes, e.g. *GDF-15*, *ATF3* and *AREG*, were found in the top 50 of three out of four exposure settings. Because of this overlap in the different exposure settings we considered these three genes plausible candidates, and selected these for validation by qRT-PCR.

**Table 1:** Top 50 genes with largest difference in expression in the different exposition settings in HMEC-1.

**BLEOMYCIN - ACUTE**		**BLEOMYCIN - CHRONIC**		**CISPLATIN - ACUTE**		**CISPLATIN - CHRONIC**	
**Gene**	**ΣZ**	**Gene**	**P-value**	**Gene**	**ΣZ**	**Gene**	**P-value**
ATF3 * ‡	↑ 9.574	CYP1B1	↑ <0.0001	CORT	↑ 9.577	MX1†	↓ <0.0001
KIAA1370	↑ 9.574	KLF12	↑ <0.0001	TREM2	↑ 9.222	GPR87	↓ <0.0001
RRAD	↑ 9.552	PRSS35 ‡	↑ <0.001	ATF3 * §	↑ 9.039	IFI27	↓ <0.0001
C12orf5	↑ 9.476	GLS2	↓ <0.001	AVPI1	↑ 8.757	JMJD4	↑ <0.001
GDF-15 ‡	↑ 9.454	ENPP4	↑ <0.001	AK1*	↑ 8.658	FAM155A	↓ <0.001
GPR87	↑ 9.454	ZMAT1	↑ <0.001	VASN	↑ 8.653	OAS3	↓ <0.001
MDM2	↑ 9.356	TMEM184C	↑ <0.001	SESN2 *	↑ 8.633	MX2	↓ <0.001
SESN2 *	↑ 9.258	ADAM12†	↑ <0.001	LRDD	↑ 8.633	HLA-F	↓ <0.001
COL7A1	↑ 9.16	RAB11FIP1	↑ <0.001	AREG§	↑ 8.633	AREG † §	↓ <0.001
ATG16L2	↑ 9.16	PTGER2	↑ <0.001	PLCD1	↑ 8.586	LOC158376	↑ <0.001
AK1*	↑ 9.16	SERPINB2	↑ <0.001	ADM	↑ 8.549	ALKBH8	↑ <0.001
FAS	↑ 8.964	SCFD2	↑ <0.01	LRRTM2	↑ 8.524	ABCC4	↑ <0.001
LIF	↑ 8.964	KRTAP4-8	↑ <0.01	DEDD2	↑ 8.457	HIST2H2BE	↓ <0.001
VWCE	↑ 8.866	C10orf136	↑ <0.01	RALGDS	↑ 8.37	RFT1	↑ <0.001
TP53INP1	↑ 8.817	HES1†	↓ <0.01	DNAJB2	↑ 8.364	LRRC38	↑ <0.001
VDR	↑ 8.811	COL1A2	↑ <0.01	MSX1	↑ 8.328	SNAI1†	↓ <0.001
C4orf18	↑ 8.768	HIST1H2BJ	↑ <0.01	MST150	↑ 8.322	C19orf42	↑ <0.01
FERMT1	↑ 8.734	RBPJL	↑ <0.01	NR4A3	↑ 8.157	LOC151171	↓ <0.01
FUCA1	↑ 8.691	CRTAC1	↓ <0.01	FDXR	↑ 8.143	SIAE	↓ <0.01
MCC	↑ 8.691	CCDC148†	↓ <0.01	ITPKA	↑ 8.143	IQGAP2	↓ <0.01
NELF	↑ 8.691	ROR1	↑ <0.01	KREMEN2§	↑ 8.143	POLA1	↑ <0.01
BTG2	↑ 8.691	EDN1	↓ <0.01	SLC31A2	↑ 8.126	PDK4	↓ <0.01
TGFBR1	↑ 8.691	TGFB2 ‡	↑ <0.01	CEACAM1	↑ 8.107	ATF3 † §	↓ <0.01
TMEM131	↑ 8.572	PRKCZ	↑ <0.01	IRF5	↑ 8.101	TNFSF10	↓ <0.01
CDH10	↑ 8.572	MARVELD2	↑ <0.01	FOXL2	↑ 8.101	DHX37	↑ <0.01
FZD2	↓ -7.775	ATF3 † ‡	↓ <0.01	VCAM1 *	↓ -8.432	TOMM40L	↑ <0.01
C3orf36	↓ -7.809	CD82	↓ <0.01	C7orf10	↓ -8.437	IFIT3	↓ <0.01
TRIB2 *	↓ -7.817	XTP3TPA	↑ <0.01	C3orf26	↓ -8.438	IFI44L	↓ <0.01
SMA5	↓ -7.835	SPTAN1	↓ <0.01	MYRIP	↓ -8.535	TNC†	↑ <0.01
NDRG4	↓ -7.869	KIAA1655	↑ <0.01	ROR1	↓ -8.56	KREMEN2 §	↓ <0.01
EBPL	↓ -7.873	DDB2	↓ <0.01	QKI	↓ -8.597	HNMT	↓ <0.01
TGFB2 ‡	↓ -7.912	SNAI1†	↓ <0.01	TRIB2 *	↓ -8.628	BRD9	↑ <0.01
MYCN	↓ -7.971	STC2	↑ <0.01	CDKAL1	↓ -8.647	BEX2	↓ <0.01
SEMA3A *	↓ -7.971	TNC†	↑ <0.01	TFPI	↓ -8.658	MGC33894	↓ <0.01
GIMAP2	↓ -7.992	LRRIQ1	↓ <0.01	LTBP1	↓ -8.664	DUSP1†	↓ <0.01
ZFP36	↓ -8.001	ASMT	↑ <0.01	PLK1	↓ -8.686	C7orf54	↓ <0.01
UBE2G2	↓ -8.044	GATA6	↑ <0.01	NAV1	↓ -8.714	ID4	↑ <0.01
RNASE1	↓ -8.069	GDF-15 † ‡	↓ <0.01	PAPPA	↓ -8.726	CCDC148†	↓ <0.01
MXD3	↓ -8.21	PCDHGA3	↓ <0.01	PIF1*	↓ -8.765	MDM2	↓ <0.01
HIST1H2AM	↓ -8.218	DDX19A	↑ <0.01	DHRSX	↓ -8.891	PRAP1	↓ <0.01
C14orf94	↓ -8.222	RARB	↑ <0.01	GMDS	↓ -8.928	FOS	↓ <0.01
PIF1*	↓ -8.261	CCND2	↓ <0.01	LAMA4	↓ -9.02	HES1†	↓ <0.01
OASL	↓ -8.265	BCS1L	↑ <0.01	DKFZP0335	↓ -9.02	ASPA	↓ <0.01
PRSS35 ‡	↓ -8.44	BIRC7	↑ <0.01	CD9	↓ -9.051	GDF-15 †	↓ <0.01
CLEC14A *	↓ -8.482	MX1†	↓ <0.01	FAT4	↓ -9.124	COL9A3	↓ <0.01
HOXD8	↓ -8.679	RERG	↑ <0.01	DOCK1	↓ -9.222	LGALS9	↓ <0.01
HIST1H1D	↓ -8.713	ADAMTS12	↑ <0.01	SMAD7	↓ -9.32	NR4A1	↓ <0.01
CLEC3B	↓ -8.866	DUSP1†	↓ <0.01	FHOD3	↓ -9.381	ADAM12†	↑ <0.01
VCAM1 *	↓ -9.16	AREG†	↓ <0.01	CLEC14A *	↓ -9.418	RNASE7	↓ <0.01
C9orf3	↓ -9.552	OSBPL1A	↑ <0.01	SEMA3A *	↓ -9.418	HHATL	↓ <0.01

* Overlapping genes in the “acute” exposure setting for both drugs,

^†^ Overlapping genes in the “chronic” exposure setting for both drugs.

^‡^ Overlapping genes in the “acute” and “chronic” exposure setting for bleomycin.

^§^ Overlapping genes in the “acute” and “chronic” exposure setting for cisplatin.

**cDNA microarray—GSEA.** Pathways enriched at a FDR ≤ 0.10 and *P* ≤ 0.025 in the GSEA are summarised in Table 2. In the “acute”-exposure setting to bleomycin, six pathways were enriched (all up-regulated), while no pathways were enriched in the “chronic” setting with the set criteria for FDR. Cisplatin exposure resulted in 12 enriched pathways in the “acute”-exposure setting (up-regulated n = 3, down-regulated n = 9) while six pathways were enriched in the “chronic”-exposure setting (all down-regulated). The ‘p53’ and the ‘Type I Diabetes Mellitus’ gene sets were enriched in three out of four exposure settings; genes included in this gene set are summarised in S1 Table.

**Table 2:** Gene Set Enrichment Analysis on gene expression profiles from HMEC-1 following “acute” and “chronic” exposure to bleomycin and cisplatin, using pathway definitions from KEGG.

	**BLEOMYCIN - ACUTE**		**CISPLATIN - ACUTE**		**CISPLATIN - CHRONIC**	
	FDR	P-value	FDR	P-value	FDR	P-value
**Cellular Processes; Cell Communication**						
Adherens junction			↓ 0.07	0.003		
Focal adhesion			↓ 0.04	<0.0001		
**Cellular Processes; Cell Growth and Death**						
p53 signalling pathway	↑ 0.001	<0.0001	↑ 0.02	<0.0001	↓ 0.1	<0.0001
**Environmental Information Processing; Signal Transduction**						
TGF-beta signalling pathway			↓ 0.05	<0.0001		
**Genetic Information Processing; Folding, Sorting and Degradation**						
Ubiquitin mediated proteolysis			↓ 0.04	0.001		
**Human Diseases**						
Endometrial cancer			↓ 0.04	<0.0001		
Cholera infection	↑ 0.10	0.005				
Type I Diabetes Mellitus	↑ 0.07	<0.0001	↑ 0.05	<0.0001	↓ 0.01	<0.0001
Neurodegerative disease					↓ 0.09	0.008
Prion diseases					↓ 0.08	0.01
**Metabolism; Carbohydrate Metabolism**						
Butanoate metabolism	↑ 0.02	<0.0001				
Glyoxylate and dicarboxylate metabolism			↓ 0.09	0.02		
Reductive carboxylate cycle			↓ 0.04	0.002		
Glycosylphosphatidylinositol(GPI)-anchor biosynthesis	↑ 0.03	0.002				
N-Glycan biosynthesis			↓ 0.08	<0.0001		
Linoleic acid metabolism	↑ 0.08	0.005	↑ 0.09	0.01		
Polyunsatyrated fatty acid biosynthesis			↓ 0.04	0.002		
**Organismal Systems; Immune System**						
Antigen processing and presentation					↓ 0.0	<0.0001
Toll-like receptor signalling pathway					↓ 0.08	<0.0001

No pathways were enriched according to these criteria after “chronic” exposure to bleomycin.

### qRT-PCR

**Figure 2:**
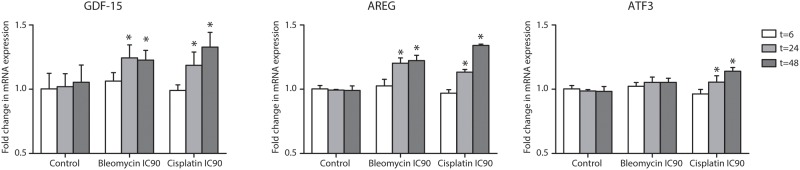
Relative differences (mean ± standard deviation) in gene expression of *GDF-15*, *ATF3*, *AREG* in HMEC-1 after “acute” exposure to bleomycin and cisplatin measured by qRT-PCR.

To validate changes in expression of *GDF-15*, *ATF3* and *AREG* qRT-PCR was performed. In the “acute”-exposure setting, mRNA-expression of all three genes increased in time after exposure to bleomycin and cisplatin, in concordance with the microarray data. After 48 hours exposure to both drugs, mRNA expression of all three genes was significantly higher compared to untreated control cells. No change in mRNA expression of these three genes occurred in untreated control cells in time (Fig. 2).

### Plasma GDF-15 protein levels in testicular cancer patients treated with BEP-chemotherapy

**Figure 3:**
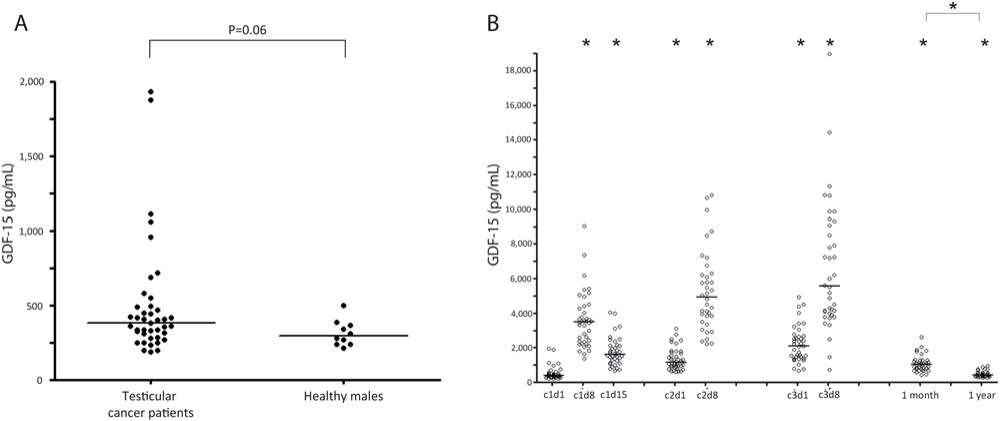
GDF-15. A. Plasma GDF-15 protein levels in patients with metastatic testicular cancer (n = 41) prior to start of bleomycin- and cisplatin-based chemotherapy, compared to healthy age-matched males (n = 10); B. Plasma GDF-15 protein levels before, during and after completion of bleomcyin- and cisplatin-based chemotherapy for testicular cancer. The sample at c1d1 is drawn before initiation of chemotherapy. (*) p < 0,05 compared to baseline value or indicated time-point.

Based on data from the literature we selected GDF-15 for further validation on the protein level in plasma of testicular cancer patient during and after treatment, and related GDF-15 levels to known plasma endothelial damage biomarkers (vWF, hsCRP) [25, 26]. Baseline characteristics of the patients are summarised in Table 3. Although short from significance (p = 0.06), baseline (i.e. before start of chemotherapy) GDF-15 protein levels in testicular cancer patients were not different from healthy age-matched males (Fig. 3A). Patients in the IGCCCG good prognosis group had slightly lower baseline GDF-15 protein levels than patients in intermediate or poor prognosis groups (good prognosis: median 362.9 pg/mL (range 197.6–1059.5; n = 33), intermediate/poor prognosis: median 689.1 pg/mL (range 186.8–1935.0; n = 8); *P* = 0.04). Median baseline GDF-15 level was not related to tumor stage (stage 2 disease: median 373.0 pg/mL (range 197.6–1935.0; n = 30), stage 3&4 disease: median 486.4 pg/mL (range 186.8–1875.9; n = 11); *P* = 0.40). Pre-chemotherapy GDF-15 protein levels were not related to age (r_s_ = 0.08; *P* = 0.61).

**Table 3:** Characteristics of 41 patients with disseminated testicular cancer and treated with cisplatin containing combination chemotherapy.

	**Median (range)**	**Number (%)**
**Number of patients**		41
**Age at start of treatment, years**	31 (18–46)	
**Diagnosis**		
Non-seminoma		35 (85.4)
Seminoma		6 (14.6)
**IGCCCG prognosis group**		
Good		33 (80.5)
Intermediate		7 (17.1)
Poor		1 (2.4)
**Tumor Stage**		
II		30 (73.2)
III		4 (9.8)
IV		7 (17.1)
**Treatment regimen**		
3 cycles BEP		29 (70.7)
4 cycles BEP		11 (26.8)
4 cycles EP		1 (2.4)

Abbreviations: International Germ Cell Cancer Collaborative Group (IGCCCG); bleomycin etoposide cisplatin chemotherapy (BEP).

During BEP-chemotherapy, GDF-15 protein levels increased compared to baseline, with significantly higher levels 1 months and 1 year post-chemotherapy (Fig. 3B, Table 4). Compared to pre-chemotherapy, plasma levels of vWF and hsCRP changed significantly during treatment (Table 4). After completion of chemotherapy, vWF-levels remained persistently elevated, whereas hsCRP returned to pre-chemotherapy values. At baseline, levels of GDF-15 were related to levels of vWF (r_s_ = 0.35; *P* = 0.03) and hsCRP (r_s_ = 0.39; *P* = 0.014). During chemotherapy, levels of GDF-15 correlated with hsCRP at c1d8 (r_s_ = 0.44; *P* = 0.01) and with vWF at c3d8 (r_s_ = 0.40; *P* = 0.017). At the follow-up visit one month after completion of chemotherapy, levels of GDF-15 and vWF were strongly correlated (r_s_ = 0.56; *P* = 0.001), whereas this relation was not found for GDF-15 and hsCRP (r_s_ = 0.18; *P* = 0.28). One year after start of chemotherapy, no relation between GDF-15 levels and vWF or hsCRP (r_s_ = 0.28; *P* = 0.12; r_s_ = 0.10; *P* = 0.57) was found.

**Table 4:** Plasma levels of GDF-15 (pg/mL), vWF (%) and hsCRP (mg/L) in testicular cancer patients (n = 41) before, during and after completion of bleomycin- and cisplatin-based chemotherapy.

	**GDF-15** (pg/mL)		**vWF** (%)		**hsCRP** (mg/L)	
	*Median*	*Range*	*Median*	*Range*	*Median*	*Range*
**Course1**						
Day 1 (= baseline)	383.1	186.8–1935.0	100	42–297	2.0	0.2–87.1
Day 8	3473.7 *	1344.5–9028.3	164*	56–319	0.6*	0.2–4.9
Day 15	1587.2 *	655.2–4014.9	145*	57–394	4.2†	1.1–101.0
**Course 2**						
Day 1	1145.5 *	593.6–3074.5	143*	34–360	5.1	1.1–39.8
Day 8	4898.0 *	2183.0–10794.9	197*	66–464	0.6*	0.2–21.1
**Course 3**						
Day 1	2067.7 *	639.0–4918.0	194*	66–440	3.7	0.3–39.9
Day 8	5542.8 *	702.3–18958.9	197*	81–419	0.6	0.2–25.7
**One month after completion of chemotherapy**						
	1009.6 *	409.7–4737.1	135*	56–249	2.2	0.4–29.1
**One year after start of chemotherapy**						
	395.2 * ‡	246.9–913.2	115* ‡	49–218	1.5§	0.2–14.3

* *P* < 0.01 compared to baseline, Wilcoxon signed rank test

^†^ *P* < 0.05 compared to baseline, Wilcoxon signed rank test

^‡^ *P* < 0.01 compared to one month after completion of chemotherapy, Wilcoxon signed rank test

^§^ *P* < 0.05 compared to one month after completion of chemotherapy, Wilcoxon signed rank test

## Discussion

In this study we used an unbiased translational approach with cDNA microarray as a tool to find novel mechanisms related to and select candidate biomarkers involved in chemotherapy-induced endothelial damage. With this *in vitro* strategy, we found several single genes with significant changes in expression upon exposure to bleomycin and cisplatin. Three genes with strong expression differences in three out of four experimental settings, *GDF-15*, *ATF3* and *AREG*, were validated by qRT-PCR. In addition, GSEA revealed clusters of genes involved in several pathways, including ‘p53’ and ‘Type I Diabetes Mellitus’ gene sets, which were affected in this model. Furthermore, we showed that BEP-chemotherapy (Bleomycin; Etoposide; Cisplatin) did indeed affect plasma GDF-15 protein levels in testicular cancer patients and that these levels related to known endothelial damage biomarkers such as vWF and hsCRP.

In the single gene analysis several genes were significantly differentially expressed in the different experimental settings in the HMEC-1 *in vitro* model. The genes *GDF-15*, *ATF3* and *AREG* were in the top 50’s of most differentially expressed genes in three or four exposure settings.

The cytokine GDF-15 (also known as Macrophage Inhibitory Cytokine 1 (MIC-1) or NSAID activated gene (NAG-1)) is a member of the Transforming Growth Factor β (TGFβ) family. GDF15 is induced by all sorts of stimuli including cytokines, chemo- and/or radiotherapy and injury in all sorts of different cells and tissues [27–30]. Release of GDF-15 can induce anti-inflammatory, anti-apoptotic and anti-proliferative effects, thereby exerting vasculoprotective mechanisms. Therefore, increases in GDF-15 plasma levels in our patient cohort may well result from increased production by endothelial cells and/or macrophages, to compensate for chemotherapy-induced damage. In a study evaluating gene expression changes in prostate cancer samples pre- and post docetaxel/mitoxantrone treatment, *GDF-15* was one of highest up-regulated genes post-treatment [31], illustrating that cytostatics can influence *GDF-15* expression in both malignant and non-malignant cells.

Elevated levels of GDF-15 have been associated with increased risk of diseases hypothesised to result from chronic inflammation [29, 32] and numerous studies showed that GDF-15 is a valuable biomarker for cardiovascular disease [29, 30, 32–34]. GDF-15 is thought to play a relevant role in cardiovascular damage responses [35], possibly through a pro-angiogenic response as was demonstrated in a pre-clinical model with HUVEC [36]. GDF-15 is also described as biomarker/predictor of albuminuria, known to reflect established micro-vascular damage [37]. In addition, several studies implicate GDF-15 in aspects of metabolic disorders e.g. insulin resistance and obesity [30, 38, 39]. Recently, higher levels of circulating MIC-1/GDF-15 were also associated with an increased risk of colorectal cancer supporting a role of chronic inflammation in the development of colorectal cancer [40].

*ATF3* is a downstream member of the MAP-kinase signalling pathway that encodes for a nuclear factor that stimulates transcription upon cellular stress. Fast up-regulation of *ATF3* is a central stress response in different endothelial cell models, induced by various noxious stimuli [41–46]. Interference with *ATF3*-levels protected cells from apoptosis induction, e.g. induced by TNFα in HUVEC [47], by cisplatin in a human glioblastoma cell line [42], or related to doxorubicin in cardiomyocytes [48]. Interestingly, immunohistochemically measured *ATF3*-expression is increased in atherosclerotic areas of human iliac arteries [43]. Few studies addressed *AREG*, a member of the epidermal growth factor receptors that plays an important role in cellular proliferation and survival. Breast cancer cells exposed to cisplatin secreted the AREG-protein over extended periods of time, i.e. up to 72 hours after exposition [49].

Based on the available data we decided to study plasma protein levels of GDF-15 in testicular cancer patients before, during and after treatment with BEP-chemotherapy, and relate changes to levels of known endothelial damage biomarkers [25, 26]. The increases in GDF-15 throughout treatment as well as post-chemotherapy may partly relate to cancer-related mechanisms, as the GDF-15 protein level wasslightly higher in patients with more advanced disease stage as measured by IGCCCG score, which may relate to release of GDF-15 from apoptotic tumour cells. However, its role in endothelial damage is supported by the fact that GDF-15 levels correlated with proteins known to reflect chemotherapy-related endothelial damage in testicular cancer patients, vWF and hsCRP [25, 26]. Therefore, it is conceivable that the observed rise in plasma GDF15 is involved in chemotherapy-related endothelial damage, however it is probably not due to endothelial injury alone. As this protein is mechanistically related to chemotherapy-related endothelial damage it may be a potentially sensitive biomarker for detecting this damage. Further extended analysis of GDF15 levels in combination with phenotyping of cardiovascular risk factors in a larger cohort of patients is warranted. Although in this study no relation was found between pretreatment levels of GDF15 and age, it is known that this is the case in the normal population and should be taken into account when analyzing GDF15 levels in time. Moreover, the fact that the TGFβ-pathway is involved in this damage may be a clue to interventions that decrease the amount of endothelial damage related to treatment with bleomycin and cisplatin.

GSEA showed significantly enriched of several pathways, including ‘p53’ and ‘Type I Diabetes Mellitus’ in “acute” exposure to bleomycin and cisplatin, and “chronic” exposure to cisplatin. The finding that p53-related genes were affected by bleomycin as well as cisplatin illustrates the validity of our approach, as both drugs exert their key therapeutic effect by cellular apoptosis induction. The enriched ‘Type I Diabetes Mellitus’ gene set includes several genes involved in inflammatory processes, e.g. Human Leucocyte Antigen-molecules, interleukins and TNF, indicating that bleomycin and cisplatin induce an inflammatory response in endothelial cells. This finding is completely in line with studies in testicular cancer patients treated with cisplatin-based regimens, which showed higher rates of systemic inflammation and endothelial dysfunction [25, 26, 50]. As circulating platinum remains detectable in the circulation years to decades after cisplatin treatment [51–52], long-term testicular cancer survivors may well have ongoing vascular damage and chronic low-grade endothelial inflammation. When chronic inflammatory responses prove to be an important pathogenic factor for the development of chemotherapy-induced endothelial damage, intervention with anti-inflammatory drugs is a rationale approach to alleviate these effects.

In conclusion, we utilised cDNA microarray to detect in an unbiased way genes and pathways associated with chemotherapy-related endothelial damage, to find biomarkers for and mechanisms involved in this damage. In HMEC-1 exposed to bleomycin and cisplatin, several genes were strongly differentially expressed, e.g. *GDF-15*, *ATF3* and *AREG*. In GSEA, clusters of genes involved in cell death and inflammation were affected. The observed changes in plasma GDF-15 protein levels in testicular cancer patients induced by cisplatin- and bleomycin-containing chemotherapy indicate that this informative pre-clinical approach can be translated to a clinical setting, and that GDF-15 may be a potential biomarker of interest that is mechanistically involved in chemotherapy-related healthy tissue damage such as endothelial damage. Further *in vitro* and *in vivo* exploration is warranted. This facilitates the rationale towards selection of targets for intervention, with early surrogate biomarkers for chemotherapy-related endothelial damage.

## Supporting Information

S1 Fig Experimental design. A. “acute” exposure setting: immortalised HMEC-1 were exposed to bleomycin (0.3 μg/ml (IC50), 1.5 μg/mL (IC90)) or cisplatin (2.6 μM (IC50), 12.9 μM (IC90)) for 6, 24 and 48 hours; B. “chronic” exposure setting: over the course of 30 days HMEC-1 was exposed to 0.06 μg/mL bleomycin (IC10) or 0.52 μM cisplatin (IC10) twice weekly). In both experiments untreated samples served as controls. (*) RNA-isolation and cDNA microarray experiments; (†) RNA isolation and qRT-PCR.
(TIF)

S1 Table Genes included in the ‘p53’ and ‘Type I Diabetes Mellitus’ gene sets in the KEGG database, ranked in alphabetical order. (DOC)
