# Supplementary figures and images for: Growth Differentiation Factor 15 (GDF-15) Plasma Levels Increase during Bleomycin- and Cisplatin-Based Treatment of Testicular Cancer Patients and Relate to Endothelial Damage
Source: PLoS One. 2015 Jan 15;10(1):e0115372. doi: 10.1371/journal.pone.0115372 (PMC4295859; doi:10.1371/journal.pone.0115372)

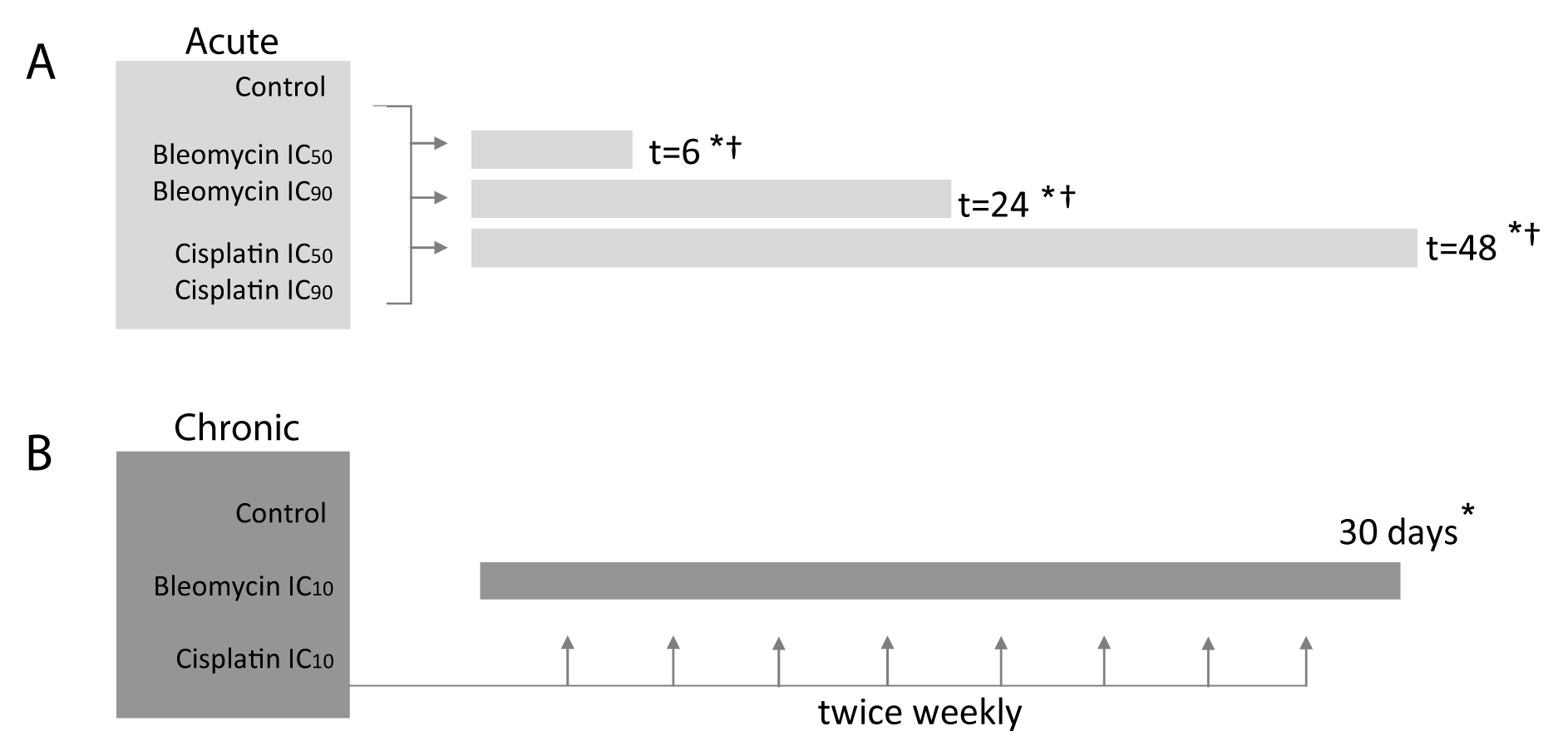

Supplement: S1 Fig — A. “acute” exposure setting: immortalised HMEC-1 were exposed to bleomycin (0.3 μg/ml (IC50), 1.5 μg/mL (IC90)) or cisplatin (2.6 μM (IC50), 12.9 μM (IC90)) for 6, 24 and 48 hours; B. “chronic” exposure setting: over the course of 30 days HMEC-1 was exposed to 0.06 μg/mL bleomycin (IC10) or 0.52 μM cisplatin (IC10) twice weekly). In both experiments untreated samples served as controls. (*) RNA-isolation and cDNA microarray experiments; (†) RNA isolation and qRT-PCR. (TIF) [file pone.0115372.s001.tif]
