# Supplementary material for: Growth Differentiation Factor 15 (GDF-15) Plasma Levels Increase during Bleomycin- and Cisplatin-Based Treatment of Testicular Cancer Patients and Relate to Endothelial Damage
Source: PLoS One. 2015 Jan 15;10(1):e0115372. doi: 10.1371/journal.pone.0115372 (PMC4295859; doi:10.1371/journal.pone.0115372)
Supplement: S1 Table — (DOC) [file pone.0115372.s002.doc]

**Table S1.**

Genes included in the ‘p53’and ‘Type I Diabetes Mellitus’ gene sets in the KEGG database, ranked in alphabetical order.

| **p53 pathway** | | **Type I Diabetes Mellitus pathway** | |
| --- | --- | --- | --- |
|  | APAF1 |  | CD28 |
|  | ATM |  | CD80 |
|  | BAX |  | CD86 |
|  | BCL2 |  | CPE |
|  | CCND1 |  | FAS |
|  | CCNE1 |  | FASLG |
|  | CDK2 |  | GAD1 |
|  | CDK4 |  | GAD2 |
|  | CDKN1A |  | GZMB |
|  | E2F1 |  | HLA-A |
|  | GADD45A |  | HLA-C |
|  | MDM2 |  | HLA-DMA |
|  | PCNA |  | HLA-DMB |
|  | RB1 |  | HLA-DOA |
|  | TIMP3 |  | HLA-DOB |
|  | TP53 |  | HLA-DPA1 |
|  |  |  | HLA-DPB1 |
|  |  |  | HLA-DQA1 |
|  |  |  | HLA-DQA2 |
|  |  |  | HLA-DQB1 |
|  |  |  | HLA-DRA |
|  |  |  | HLA-DRB5 |
|  |  |  | HLA-E |
|  |  |  | HLA-F |
|  |  |  | HLA-G |
|  |  |  | ICA1 |
|  |  |  | IFNG |
|  |  |  | IL12A |
|  |  |  | IL12B |
|  |  |  | IL1A |
|  |  |  | IL1B |
|  |  |  | IL2 |
|  |  |  | INS |
|  |  |  | LTA |
|  |  |  | PRF1 |
|  |  |  | PTPRN |
|  |  |  | PTPRN2 |
|  |  |  | TNF |
